# Modeling the extinction risk of European butterflies and odonates

**DOI:** 10.1002/ece3.9465

**Published:** 2022-11-08

**Authors:** Sophia Franke, Stefan Pinkert, Roland Brandl, Simon Thorn

**Affiliations:** ^1^ Department of Animal Ecology, Faculty of Biology Philipps‐Universität Marburg Marburg Germany; ^2^ Department of Conservation Ecology, Faculty of Biology Philipps‐Universität Marburg Marburg Germany; ^3^ Hessian Agency for Nature Conservation, Environment and Geology State Institute for the Protection of Birds Gießen Germany

**Keywords:** European red list, freshwater, phylogenetic signal, terrestrial, trait‐based analyses

## Abstract

Insect populations have become increasingly threatened during the last decades due to climate change and landuse intensification. Species characteristics driving these threats remain poorly understood. Trait‐based analyses provide a straight‐forward approach to gain a mechanistic understanding of species' extinction risk, guiding the development of conservation strategies. We combined morphological traits and phylogenetic relationship for 332 European species of butterflies and 115 species of odonates (dragon and damselflies) to model their red list status via phylogenetically controlled ordered logistic regression. We hypothesized that extinction risk increases with increasing body volume and wing area, decreasing range size, and is larger for brighter species. All investigated traits exhibited a strong phylogenetic signal. When controlling for phylogenetic relationship, we found that extinction risk of butterflies increased with decreasing range size. The extinction risk of odonates showed no relationship with the selected traits. Our results show that there is no universal trait defining the extinction risk of our investigated insect taxa. Furthermore, evolutionary history, measured as the phylogenetically predicted part of our analyzed traits, poorly predicted extinction risk. Our study confirms the focus of conservation measures on European butterfly species with small range sizes.

## Graphical abstract

A trait‐based analysis for European butterflies and odonates to gain a mechanistic understanding of species’ extinction risk. We found no universal trait defining the extinction risk of our investigated insect taxa. When controlling for phylogenetic relationship the extinction risk of butterflies increased with decreasing range size while extinction risk of odonates showed no relationship with the selected traits.

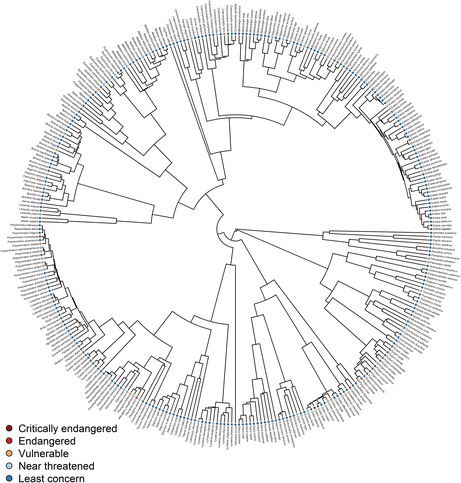

## 1 INTRODUCTION

Insects are an important part of the world's terrestrial and freshwater biodiversity (Mora et al., 2011). They provide crucial ecosystem services, such as decomposition and pollination (Cardoso et al., 2020; Macadam & Stockan, 2015; Santos et al., 2020). During the last decades, a lot of insect populations became threatened due to pollution, climate change, and land‐use intensification (Clausnitzer et al., 2009; Warren et al., 2021). This has resulted in declining abundances, local extinctions, and reduction of overall insect biomass (Basset & Lamarre, 2019; Seibold et al., 2019). These effects are particularly strong in landscapes dominated by agriculture, whereas they are less pronounced in freshwater ecosystems (van Klink et al., 2020). In some regions, odonates even rapidly recover under favorable environmental conditions (Termaat et al., 2015). Both habitat loss and the loss of connectivity between the remaining habitats contribute to insect decline (Habel, Samways, & Schmitt, 2019; Habel, Ulrich, et al., 2019). Another important threat is climate change, especially its consequences on freshwater biodiversity due to, e.g., an increasing anthropogenic water demand (Koutroulis et al., 2018; Vörösmarty et al., 2010).

The threat of species can broadly be divided into extrinsic and intrinsic factors. Former are defined as factors describing the environment in which a species lives, including but not limited to habitat change, land‐use, and climate change. The latter factors refer to traits of species that determine their interaction with the environment, such as physiological adaptations as well as traits involved in resource use or dispersal (Seibold et al., 2015). For instance, body size is correlated with resource use (Pinkert et al., 2020; Savage et al., 2004), metabolism (Gillooly et al., 2001), development rates (Gillooly et al., 2002), and population densities (Pinkert et al., 2020). Large species require more energy and are therefore characterized by smaller populations within a given habitat. Hence, species with larger body sizes are more likely to be threatened due to demographic stochasticity (Lande et al., 2003; Melbourne & Hastings, 2008) than their smaller relatives (Fritz et al., 2009; Gaston & Blackburn, 1995; Suárez‐Tovar et al., 2019). Wing size is a proxy for the dispersal ability and in a wider context for predicting the range size, being therefore crucial for the extinction risk since mobile species are less endangered than less mobile ones (Outomuro & Johansson, 2019; Pöyry et al., 2009; Sekar, 2012). Coloration also corresponds to several aspects of environmental interaction such as fitness and distribution range (Clusella Trullas et al., 2007; Pinkert et al., 2020).

Butterflies and odonates (damselflies and dragonflies) are charismatic insect groups that are among the most intensively studied insect lineages (Kalkman et al., 2018; Lewis & Senior, 2011). Both groups are easy to identify, a fact that predestines these two groups as indicators for biodiversity changes in terrestrial (Thomas, 2005) and freshwater ecosystems (Dolný et al., 2013; Miguel et al., 2017). Yet, extrinsic extinction factors such as habitat fragmentation and water scarcity have been repeatedly studied for these groups (Kalkman et al., 2018; Thomas, 2016), mainly in Central and Northern Europe (Tang & Visconti, 2021). However, much less is known for traits increasing the extinction risk of species and whether trait–threat relationships are similar across taxa (Nylin & Bergström, 2009).

We used data on European butterflies and odonates as representatives of terrestrial and freshwater insects. We tested (i) whether larger species are more endangered than smaller ones using body size, (ii) whether widely distributed species are less endangered than locally distributed ones (using wing area or the gridded distribution across Europe), whether (iii) darker colored species are less endangered than lighter colored ones, and (iv) to what extent the results are influenced by evolutionary relationships.

## 2 MATERIAL AND METHODS

### 2.1 Species data

The taxonomy and nomenclature of European butterflies were taken from Wiemers et al. (2018). To estimate extinction risk, we used the European Red List of Butterflies from 2010 (van Swaay et al., 2010). Totally, 451 species were assessed within this list. Our analyses relate to the EU 27 countries. About 7% of butterflies occurring within the EU 27 countries are threatened (9% for Europe) and an additional 10% are considered as near threatened (van Swaay et al., 2010). The recent European Red List of odonates from 2010 contains 137 assessed species. Of these, 15% are listed in one of the three IUCN threat categories (Kalkman et al., 2010). We excluded species endemic to small islands and species that reach Europe with their distributional edges (e.g., distributed mainly in Asia). In general, we restricted our analysis to species with retrievable red‐list status, phylogeny, and traits. This resulted in complete data sets for 332 butterflies and 115 odonates.

To assess the relationship between species' traits and their extinction risk as estimated by the red list status, we selected four different morphological and biogeographical traits, namely body volume in cm^3^ as a measure of body size, color lightness via the additive color mixing with the basic colors red‐green‐blue (mean RGB value), wing area in cm^2^, and the geographical range size. These traits were available for both butterflies and odonates. We followed Pinkert et al. (2018) and Zeuss et al. (2014) using drawings of European butterflies (Tolman & Lewington, 2009) and of European odonates (Dijkstra & Lewington, 2006) to estimate body size and color lightness. To prepare images for the analysis, the body (head, abdomen, and thorax) in scanned drawings of species' dorsal body surfaces (24‐bits, sRGB, 1200 dpi resolution) was cropped out and saved to separate files using functions of Adobe Photoshop CS2.

Based on these images, we calculated the body volume in cm^3^ (*π* × [½ length of pixel row]^2^ × pixel edge length) as an estimate of the body size of a species based on the assumption that bodies of butterflies and odonates generally have a cylindrical form. The calculations were performed using functions of the R‐package *png* (Urbanek, 2013). Body volume instead of linear size measures, such as wing length, head width, and body length, was used because it allows for a more realistic estimate of the body mass as a three‐dimensional measure of a body size (Kühsel et al., 2017). Note that previous studies have shown that the color lightness and body volume estimates are correlated between drawings from different sources and between males and females (Pinkert et al., 2017; Zeuss et al., 2017).

In addition, we calculated the average color of pixels of an image across the red, green, and blue channels (RGB) as an estimate of the color lightness of a species (8‐bit gray values ranging from 0: absolute black to 255: pure white). For estimating the color lightness of butterfly species, we focused on the body and 1/3 of the wing area closest to the body because this area is probably the most important for thermoregulation (Tsai et al., 2020; Wasserthal, 1975). For example, in the *Pieridae* family, most species have white wings, but with a dark wing base, which is linked to the V‐wing position basking behavior. Some butterfly species use lateral basking for thermoregulation (e.g. Satyrinae), whereas dorsal and ventral color estimates are correlated (Zeuss et al., 2014). Differences between clades are considered regarding phylogeny.

The wing area was calculated as the number of pixels of the four wings × pixel area in cm^2^. To obtain a measure of species' dispersal abilities, wing area estimates were corrected for body size considering only the residual variation in wing area from a linear regression between the log‐transformed (natural logarithm) wing area and the log‐transformed (natural logarithm) body volume (Outomuro et al., 2021).

Gridded distribution data on European butterflies was taken from Schweiger et al. (2014). For odonates, we intersected vector distribution maps (Dijkstra and Lewington (2006)) with a grid of equal area cells (~50 km × 50 km). As an estimate of range sizes, range occupancies were calculated as the number of grids occupied by a species relative to the total number of grid cells covering Europe. Furthermore, we compiled the flight period length (i.e. the sum of months when the imago is active) and the annual mean temperature of occupied grid cells (Karger et al., 2017), but excluded them from the final modeling due to multi‐collinearity. Multicollinearity was assessed using the function *vifcor* of the R‐package *usdm* (Naimi et al., 2014).

### 2.2 Statistical analyses

All statistical analyses were performed in R version 4.0.2 (R Core Team, 2020). The phylogenetic relationship of species violates the statistical requirement of independent observations (Felsenstein, 1985). Hence, we corrected species traits by their respective phylogenetic relationship among each other. To do so, we used the phylogeny of European odonates from Pinkert et al. (2018) and the phylogeny of European butterflies from Wiemers et al. (2020). Both phylogenies were constructed using a Bayesian framework that integrated morphological as well as phylogenetic data, and they are fully resolved to the species level. Given these and other similarities, the quality of the two phylogenies is comparable. First, we tested the selected traits for their phylogenetic signal using Pagel's lambda, a value between zero (no signal) and one, using the function *phylosig* of the R‐package *phytools* (Revell, 2012). We controlled traits if they had a significant phylogenetic signal. Subsequently, we decomposed each trait into its phylogenetically predicted part (ancestral component of the trait, hereafter P‐component; Lynch's A + u) and the residual deviation (species‐specific variance of the trait, hereafter S‐component; Lynch's E) using Lynch's comparative method (Lynch, 1991).

To relate the species traits to their extinction risks, we used threat categories of the European Red List. These were converted into an ordinal scale of extinction risk ranging from 0 (Least Concern) to 4 (Critically Endangered). Ordinal scaled data require ordinal regression models (Seibold et al., 2015; Verde Arregoitia et al., 2013). We tested for relationships between species' traits and their extinction risk using an ordered logistic regression with the function *polr* of the R‐package *MASS* (Agresti, 2010) with the red list status as ordered factor response variable and the traits body volume, wing area, and color lightness, as predictors. The assessment of the red‐list status is in part based on the species range size. Hence, we included the geographical range size as a predictor to statistically account for this effect. This modeling approach estimates the relative strength of predictors in determining a species' extinction risk, controlled by range size.

## 3 RESULTS

All traits had a significant phylogenetic signal (Table 1). For butterflies, the strength of the phylogenetic signal decreased from body volume to wing area, to color lightness, and to range size. Body volume and range size of odonates had the highest phylogenetic signal and color lightness the lowest. A significant phylogenetic signal necessitates a control for the phylogeny in the further statistical modeling.

**TABLE 1:** Phylogenetic signal lambda of all four traits for 332 butterflies and 115 odonates calculated via the phylosig function (phytools).

	Butterflies	Odonates
	Phylogenetic signal λ	Phylogenetic signal λ
Body volume	0.99	1.00
Wing area	0.93	0.94
Color lightness	0.89	0.76
Range size	0.27	1.00

*Note*: In all cases *p* < .001.

The phylogenetic component of the selected traits did not influence the extinction risk of our studied groups (Table.2, P—component). The same applies for the species‐specific component and the extinction risk of odonates. However, analyzing the S‐component, the extinction risk of butterflies decreased with increasing range size. Body volume and wing area had no significant influence on the extinction risk of both groups (Table 2).

**TABLE 2:** Effects of body volume, wing area, color lightness, and range size on the extinction risk of 332 European butterflies and 115 odonates modeled by ordered logistic regression.

Traits	Butterflies				Odonates			
***S ‐ component***	*Estimate*	*Std.error*	*z‐value*	*p‐value*	*Estimate*	*Std. error*	*z‐value*	*p‐value*
Body volume	−17.06	27.51	−0.62	.54	6.59	58.39	0.11	.91
Wing area	−1.08	2.82	−0.38	.70	10.51	8.88	1.18	.24
Color lightness	−0.01	0.23	−0.42	.67	0.08	0.04	1.79	.07
Range size	−0.71	0.22	−3.09	**.01**	9.47	44.26	0.21	.83
***P – component***	*Estimate*	*Std.error*	*z‐value*	*p‐value*	*Estimate*	*Std. error*	*z‐value*	*p‐value*
Body volume	1.20	2.01	−0.60	.55	−3.48	4.07	−0.86	.39
Wing area	0.36	0.49	0.73	.46	−1.96	1.44	−1.36	.17
Color lightness	0.003	4.64 x10^−3^	0.61	.54	0.01	0.02	0.32	.75
Range size	−0.28	0.49	−0.57	.57	1.92	1.92	1.00	.32
**Raw data**	*Estimate*	*Std.error*	*z‐value*	*p‐value*	*Estimate*	*Std. error*	*z‐value*	*p‐value*
Body volume	1.19	1.77	0.67	.50	−1.33	2.93	−0.45	.65
Wing area	0.42	0.47	0.91	.36	−0.92	1.19	−0.78	.44
Color lightness	2.16 × 10^−4^	4.04 × 10^−3^	0.54	.59	0.02	0.02	1.30	.20
Range size	−0.52	0.19	−2.73	**.01**	.96	1.39	0.70	.49

*Note*: The P‐component represents the phylogenetically predicted part of the respective trait, and the S‐component represents the respective deviation of the average trait from the P‐component. The raw data represent the trait values without phylogenetic control. Significant relationships (*p* < .05) are given in bold.

## 4 DISCUSSION

Our study showed that the extinction risk of butterflies increased with decreasing range size (Table 2). Thereby, the extinction risk of butterflies only depends on the species‐specific variation of range size and not on the phylogenetically predicted part of this trait. The extinction risk of European odonates showed no relationship with the selected traits (Table 2).

**FIGURE 1:**
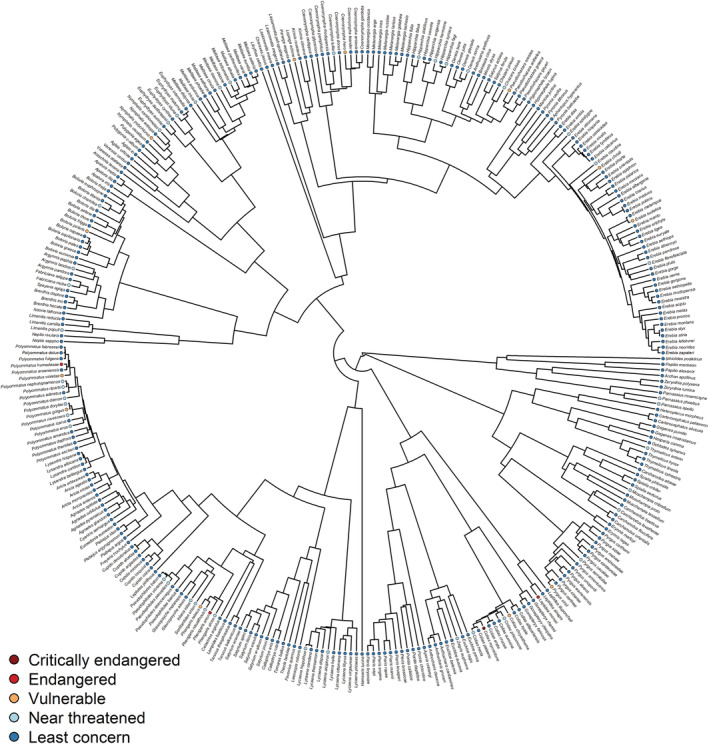
Phylogenetic tree of 332 European butterfly species and their IUCN red list status

**FIGURE 2:**
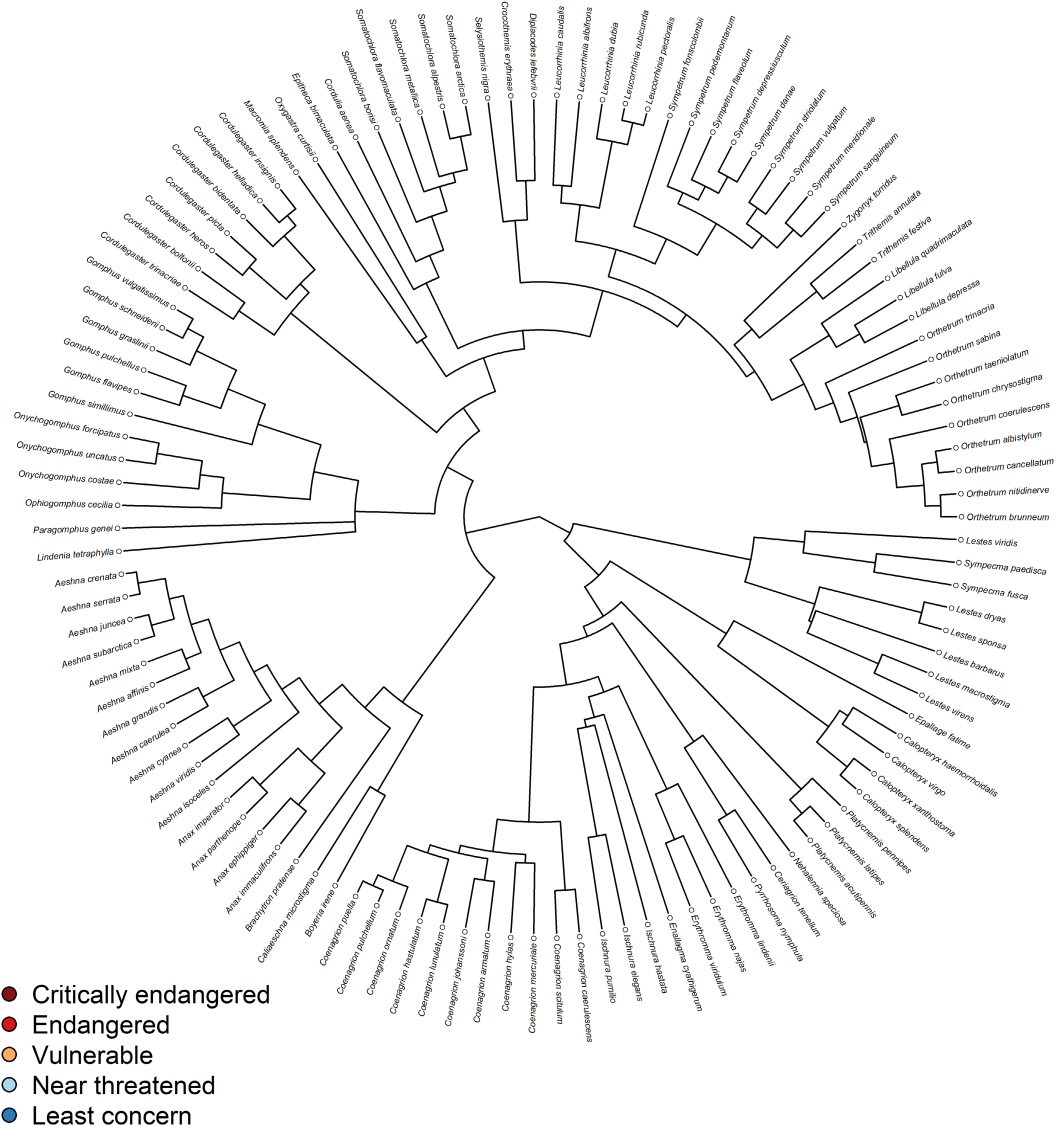
Phylogenetic tree of 115 European odonates and their IUCN red list status

In our study, all traits exhibited a strong phylogenetic signal (Table 1). The phylogenetic signal quantifies the tendency of related species to resemble each other more than species randomly drawn from the same phylogenetic tree (Blomberg et al., 2003) (Figure 1, Figure 2). Despite this fundamental fact, Chichorro et al. (2019) found that only half of the 24 evaluated studies on insects were phylogenetically controlled. We split the variance in our morphological traits into phylogenetic and species‐specific components to determine the influence of the respective component on our analyses. In this case, the phylogenetic component showed no influence, but the species‐specific component, which is thought to represent recent adaptations to climatic conditions (Pinkert et al., 2017), niche conservatism and dispersal limitations (Pinkert et al., 2018), did.

Studies investigating moths commonly do not control species traits in a consistent manner for evolutionary ancestry, often caused by the absence of valid molecular phylogenies (Nieminen et al., 1999; Slade et al., 2013). Coulthard et al. (2019) found, via a genetic distance matrix, no significant relationship between phylogenetic relatedness and population trends, but traits in general as reliable predictors of population changes in moths. Further, they highlight that the relationships of life‐history traits are not always in line with conclusions drawn from literature (Coulthard et al., 2019). Two further measures to evaluate the phylogenetic signal are Blomberg's K and Pagel's λ. All studies that calculated these measures determined a high and significant phylogenetic signal in the studied insect traits (Arbetman et al., 2017; Arnan et al., 2017; Pinkert et al., 2020; Suárez‐Tovar et al., 2019). We used Pagel's λ to quantify the strength of the phylogenetic signal in our investigated traits (Diniz‐Filho et al., 2012; Freckleton et al., 2002; Pagel, 1999).

Our result that wider distributed butterfly species were less prone to extinction (Table 2) is in line with other studies that analyzed the role of species distribution for extinction risk (Arbetman et al., 2017; Korkeamäki & Suhonen, 2002; Mattila et al., 2008; Pöyry et al., 2009). Butterflies with narrow distribution ranges are more prone to extinction, such as, e.g. *Pseudochazara orestes* and *Polyommatus humedasae* (Habel et al., 2020; Maes et al., 2019). Additionally, forest macro moth species can be threatened by decreasing forest connectivity, despite their high dispersal capability (Slade et al., 2013). However, we did not analyze traits connected to habitat use, which would enable a more direct link to anthropogenically altered habitats and resources (Seibold et al., 2015). In contrast to the butterfly model, for odonates the influence of range size on the extinction risk was not significant. This result is remarkable because the correlation of range size and extinction risk seems trivial and was found in several insect groups (Mattila et al., 2006; Terzopoulou et al., 2015). However, odonates are able to use small habitat patches to overcome adverse conditions, show rapid responses to changing environment, and some species are highly mobile. For example, Kortello and Ham (2010) studied *Argia vivida* in fuel management areas and found that maintaining unmodified stands of dense trees in association with cleared patches of appropriate dimension is a valuable conservation measure for this species. Flenner and Sahlén (2008) studied community reorganization of odonates in boreal forest lakes under climate change. They found rapid reactions of the population with an equal number of species but a reduction of diversity within 10 years. Suhling et al. (2017) studied long‐distance dispersal events of odonates in arid Namibia, where individuals covered distances of several hundred kilometers without any possible reproduction habitat in between. This enables some odonates to strongly recover (Termaat et al., 2015) and might in general lead to a reduced impact of range size on the extinction risk.

Body size did not significantly influence the extinction risk of our study taxa (Table 2). Nonetheless, many studies, mainly for mammals or birds, found this correlation and one explanation is that larger species have higher viability costs, which makes them more prone to extinction (Fritz et al., 2009; Gaston & Blackburn, 1995). This general link was also found for butterflies*,* with body size measured as the median of male and female forewing length corrected by their phylogenetic relationship (García‐Barros, 2000). Although body size is often an appropriate surrogate for extinction risk, it also correlates with other traits and is, therefore, often difficult to interpret (Bennett & Owens, 1997; Chichorro et al., 2019). Suárez‐Tovar et al. (2019) studied the relation of body size and extinction risk in damselflies (Zygoptera) by measuring four components of body size (body length/head width/length of fore and hind right wings) and modeled them with a super‐tree of Zygoptera species. They found an increased risk of extinction with increasing body size for most size estimators, but not for body length, which we also found after controlling for phylogeny (Table 2). Further, it is essential that measure was taken as proxy for the body size. We selected body volume to study the influence of body size on the extinction risk and used the wing area as proxy for the dispersal ability. Wingspan is an easily accessible species‐specific trait among different taxa and can, carefully interpreted, indicate dispersal ability, but it might not be the best for trait analysis (Sekar, 2012). Bowden et al. (2015) found decreasing wing lengths as a response to warmer summers during a period of 18 years in high arctic butterflies. This in turn influences dispersal capacity and fecundity and might predispose these species to a higher extinction risk (Bowden et al., 2015). In line with our findings, Koh et al. (2004) found for males and females that body size did not affect extinction risk of tropical butterfly species (Table 2). Kuussaari et al. (2014) studied the influence of body size of butterflies, measured as the average female wingspan, on their mobility and found that, after correcting for phylogeny, the effect was not significant anymore, which is also in line with our findings (Table 2). Both studies explain this by the small variation in relative body size compared to other taxa.

## 5 CONCLUSION

In summary, our results show that intrinsic traits alone are poor predictors of the extinction risk of odonates and butterflies, despite well‐known mechanistic links of these traits to the environment and species' population dynamics. In addition, European butterfly species with smaller ranges are more vulnerable, while range size did not affect the extinction risk of European odonates. Thereby, our results underline the previous finding that improvements of the water and habitat quality have generally led to the recovery of many freshwater insects, whereas land use continues to threaten terrestrial insect diversity (Engelhardt et al., 2022). Our analyses do not support trait—extinctions risk relationships documented for well‐studied taxa such as birds and mammals, but rather suggest idiosyncratic responses of insect species to pollution, land use and climate change. Hence, both the relative importance of major threats and the mechanisms linking intrinsic traits to environmental factors need to be assessed to understand extinction of insects. Without such species‐specific information, it will be difficult to mitigate their threat.

## AUTHOR CONTRIBUTIONS

**Sophia Franke:** Conceptualization (equal); formal analysis (equal); writing – original draft (lead); writing – review and editing (equal). **Stefan Pinkert:** Conceptualization (equal); data curation (lead); writing – review and editing (equal). **Roland Brandl:** Conceptualization (equal); writing – review and editing (supporting). **Simon Thorn:** Conceptualization (equal); methodology (equal); writing – original draft (supporting); writing – review and editing (equal).

## FUNDING INFORMATION

Open Access funding enabled and organized by Projekt DEAL.

## Data Availability

All data supporting the analysis can be requested from the correspondence author and are available on Dryad; Trait data of butterflies and odonates; Phylogenetic trees of butterflies and odonates.
